# Characterization of the volatile compounds in tea (*Camellia sinensis* L.) flowers during blooming

**DOI:** 10.3389/fnut.2024.1531185

**Published:** 2025-01-14

**Authors:** Xiangyang Guo, Lulu Wang, Xiuting Huang, Qiying Zhou

**Affiliations:** ^1^College of Tea and Food Science, Xinyang Normal University, Xinyang, China; ^2^College of Chemistry and Environmental Engineering, Shenzhen University, Shenzhen, China; ^3^Dabie Mountain Laboratory, Xinyang, China; ^4^College of Horticulture, Shanxi Agricultural University, Taigu, China

**Keywords:** tea (*Camellia sinensis*) flower, volatile compounds, aroma profiles, aroma character impact (ACI), blooming stage

## Abstract

Tea flower, with characteristic flavor formed during blooming, are a significant tea resource. However, studies on the volatile compounds of tea flower and their aroma characteristics during flowering are scarce. In this study, the odor characteristics of tea flower during blooming were comprehensively investigated by GC–MS, PCA, ACI determination and sensory evaluation. The tea flower of unopened buds (TF-S1) contained the highest alcohol amounts, while fully opened tea flowers (TF-S3) had the highest heterocyclic compounds. Half-opened tea flowers (TF-S2) had the most volatile compounds, including high levels of linalool and its oxides, and low levels of (*Z*)-3-hexen-1-ol. Acetoin and cosmene were first identified in TF-S1 and TF-S2, respectively. The major ACI components differed, with linalool being prominent exhibiting ACI above 27 in all samples. Acetophenone, unique to TF-S2 with ACI of 57.35, contributed to sweet odor. Furthermore, PCA analysis and sensory evaluation revealed distinct aroma characteristics among the samples. Overall, TF-S2 and TF-S3 had higher volatile amounts and better aroma properties with floral, powdery or almond-like odors. These results advance the understanding of aroma properties of tea flower during blooming, and provide a reference for resource utilization and promotion of the application in food or cosmetics industries.

## 1 Introduction

The tea flower, derived from *Camellia sinensis* (L.) O. Kuntze, is a reproductive organ of the tea plant and constitutes an edible portion of the tea bush. It has increasingly become the second tea resource after tea leaves, which can be used to make scented teas and beverages (1). Studies have shown that tea flowers have similar bioactive compounds to those found in tea leaves, which contains polyphenols, caffeine, polysaccharides, amino acids, 2-ketobutyric acid, phenolamides (2–4). These compounds contribute to various health-promoting benefits, including antioxidant, anti-cancer, anti-inflammatory, anti-cholesterol, antifungal, hypoglycemic, hypotensive, immunomodulatory, improvement of dermal hyperpigmentation and maintaining intestinal health (5–14). Regarding the excellent biological activities, tea flowers have been paid more attentions in health products, and hold potential for application in the development of functional foods and medicines over recent years (15, 16). On the other hand, tea flower is renowned for its aroma properties, characterized by pleasant floral, fragrance, fresh, fruity and sweet odors (17), and is rich in active ingredients, such as tea polyphenols and amino acids, making it an excellent candidate for beverage production (18), particularly enhancing the fruitiness of kombucha (19). Additionally, research by Khat-Udomkiri et al. (20) highlights the potential of tea flower extract in cosmetic and pharmaceutical applications, as it effectively protects fibroblast cells from oxidative damage. To date, the majority of research has concentrated on the health benefits *in vitro* and the analysis of non-volatile secondary metabolites of tea flowers (21, 22), while comparatively fewer studies have investigated their volatile compounds and aroma characteristics.

Aroma is a crucial quality aspect of flowers or products produced thereform, affecting their sensory characteristics and economic value (23). Flowers exhibit different aroma profiles due to the presence of diverse volatile compounds, which find applications in the food flavoring and cosmetic industries (24). Furthermore, the quality and market value of tea flower related products are significantly influenced by the presence of volatile compounds. The aroma profile of tea flower is a critical factor in determining its market appeal and consumer acceptance (25). A pleasant and natural aroma can enhance the user experience and contribute to the perceived efficacy of the product, thereby increasing its market value. Notably, certain volatile compounds may also possess antimicrobial or anti-inflammatory effects, showing additional skin benefits, which can further boost the market value of tea flowers (26).

The volatile compounds in flowers are mainly formed during blooming (50), and those formed at different flowering stages possess distinct aroma characteristics. However, the volatile compounds in tea flowers, either in volatile compositions, their variations or aroma profiles at different blooming stages, have been rarely studied and reported so far. It would greatly affect the comprehensive development and utilization of tea flowers. Notably, tea flowers have been assigned as a new food resource in China, where they are abundant, with an annual output exceeding 1.8 billion kilograms (14). Given their potential as a food source and the lack of research on their aroma characteristics, it is crucial to analyze the volatile compounds and aroma profiles of tea flowers at different flowering stages. This research will not only contribute to a better understanding of tea flower aroma development but also support the comprehensive development and utilization of this abundant resource.

Headspace (HS) extraction is a convenient and straightforward method to extract aroma compounds and does not produce artifacts during operation. This method is commonly employed for extracting and enriching volatile compounds, including those found in flowers (27). Thereafter, the gas chromatography–mass spectrometry (GC–MS) is applied for the separation and analysis of volatile compounds in complex matrix (28, 29). In the current study, the aroma compounds in tea flowers were extracted using HS and analyzed by GC–MS to reveal their aroma compositions and odor profiles at different blooming stages, aiming to provide scientific foundation for the development and resource utilization of tea flowers, ultimately enhancing the overall quality of related products.

## 2 Materials and methods

### 2.1 Reagents and materials

**Figure 1:**
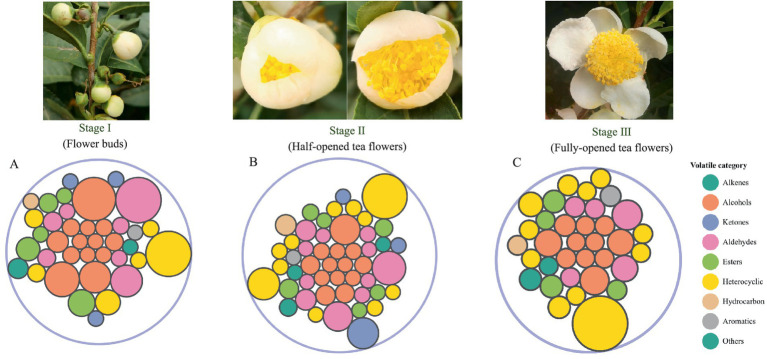
The identified volatile compounds in TF-S1 **(A)**, TF-S2 **(B)** and TF-S3 **(C)**. The bubble diagram of volatile compounds, where circles of identical colors signify compounds belonging to the same volatile category, and the size of each circle corresponds to their relative amount.

The fresh tea flowers (*Camellia sinensis* cv. Population) of three different flowering stages, which were of flower buds (stage I, unopened buds, TF-S1), half opened tea flowers (stage II, including the ones that petal started to split, TF-S2) and fully opened tea flowers (stage III, TF-S3). The detailed descriptions and states of tea flowers from three flowering stages were provided in Supplementary material and illustrated in Figure 1. These tea flower samples were collected at Guangde City (Anhui province, China) in October 2019, then immediately deep frozen in liquid nitrogen and stored in a −80°C ultra-low freezer before processing. The tea flower samples were freeze-dried using freeze dryer (ALPHA 1–4 LD plus, Martin Christ Freeze Dryer, Germany) and powdered to pass through a 350 μm sieve. All authentic standards were obtained from Sigma Aldrich (Shanghai, China) unless specified otherwise. The detail information of reference compounds and their suppliers are shown in Supplementary Table S1. A mixture of C5-C28 *n*-alkanes were purchased from Supelco, United States.

### 2.2 Volatile extraction and detection by HS-GC–MS

Volatile compounds in tea flowers were extracted by HS using a method similar to that reported previously (30) with some modification. Three grams of dried tea flowers were directly placed into a 20 mL headspace vial, and covered by a headspace auto-loading crimp cap with septa. Volatile compounds were analyzed on a GC (7890A, Agilent, Santa Clara, CA, United States) coupled to a headspace injector (Agilent 7697A, Agilent, United States) with splitless mode. The oven temperature, manifold temperature, and transfer-line temperature were set at 120, 140, and 150°C, respectively. The incubation and injection time were 20 min and 60 s, respectively. The temperature program was initially set at 50°C, held for 5 min, increased to 210°C at a rate of 3°C min^−1^ and retained 3 min, raised to 230°C at 10°C min^−1^ and subsequent maintained at 230°C for 2 min, finally increased to 280°C at a rate of 10°C min^−1^ and held for another 10 min. Injection volume was 1 μL.

The GC–MS analytical procedure for the analyzing volatile compounds in tea flowers, as described by Guo et al. (31) were followed, with minor modification. Separation was achieved by a fused-silica capillary column (DB-5, 30 m × 0.25 mm, 0.25 μm film thickness, J&W Scientific, Folsom, CA, United States). Helium gas (1 mL/min) was used as a mobile phase. The mass spectrometer was operated in electron ionization mode (positive ion, 70 eV). Ion source and transfer-line temperatures were 250 and 150°C, respectively. The detection electric pressure was 1905 V, the mass spectra were acquired in full scan mode from 30 to 500 amu. Retention indices (RI) were calculated from the retention time of *n*-alkanes by linear interpolation. The peak was deconvoluted and the compounds were identified by comparing the mass spectral data with those of the NIST virtual library (NIST 2017 version), references and in-house database (30–33). Components were tentatively identified by agreement of their retention times, retention index and mass spectra with published data and, if available, positively identified with those of authentic compounds. The amounts of the identified volatile compounds were expressed as the average of the peak area of three replicates.

### 2.3 Calculation of aroma character impact

Aroma character impact (ACI), which was the percentage of the ratio of the concentration of a volatile compound to its odor threshold value, was applied to compare aroma contributions of the identified volatile compounds in a mixture (34), which was determined by the Equation 1:


(1) $$ACI\;\left(\%\right)=\frac{\frac{C_{i}}{T_{i}}}{\sum_{i}\frac{C_{i}}{T_{i}}}\;\times 100=\frac{\frac{Ra_{i}}{T_{i}}}{\sum_{i}\frac{Ra_{i}}{T_{i}}}\;\times 100$$

where *C_i_* is the concentration of the volatile compound and *T_i_* is its corresponding odor threshold value in air; *Ra_i_* is the relative amount (peak area) of the volatile compound in tea flowers during flowering.

### 2.4 Sensory evaluation

The method of sensory analysis on tea flower aroma was from Guo et al. (33) with modification. The tea flower samples from different stages of flowering were subjected to a sensory test. Three-digit numbers were used to code samples, and they were randomly offered to panelists, the intensity values and aroma descriptors of samples were recorded by evaluators. The aroma attributes, including floral, powdery, green, citrus, waxy, fragrance, and almond-like odor were recorded by panelists. As for the TF-S2, the green odor was noted following by the floral odor, which was a little different from the green flavor of TF-S1. The intensities of the aroma attributes were scored using a scale from 0 to 10, the higher the score, the stronger the intensity, where 0 = none or not perceptible intensity, 3 = weak intensity, 5 = moderate intensity, 7 = high intensity, and 10 = extremely high intensity. Each sample was evaluated three times by each panelist on different days. Data were expressed as mean values.

The tea flower samples were evaluated by a well-trained panel of 10 members (four males and six females, age from 27 ~ 45). All assessors had more than 5 years of experience in the descriptive sensory analysis of natural plant and flower related products. Panelists were trained by a series of important volatile compounds, including (*E*)-2-pentenal for its fruity odor, (*Z*)-3-hexenal and hexanal for their green and grassy scents, (*E*,*E*)-2,4-nonadienal for its fatty odor, phenethyl alcohol, linalool and its oxides for their floral fragrances, phenylacetaldehyde for its honey-like odor, *α*-terpineol for its woody odor, maltol for its sweet odor, as well as the flavor of actual products such as almonds.

### 2.5 Statistical analysis

All experiments were replicated 3 times. Statistical analysis was performed with one-way analysis of variance (ANOVA) and Duncan’s multiple-range tests by use of SPSS Statistics 22.0 (SPSS Inc., Chicago, IL, United States). All comparisons were considered statistically significant if *p*-value <0.05. In addition, the circular stacking diagrams were generated using Chiplot.^1^

## 3 Results and discussion

### 3.1 Volatile compositions of tea flowers

**Figure 2:**
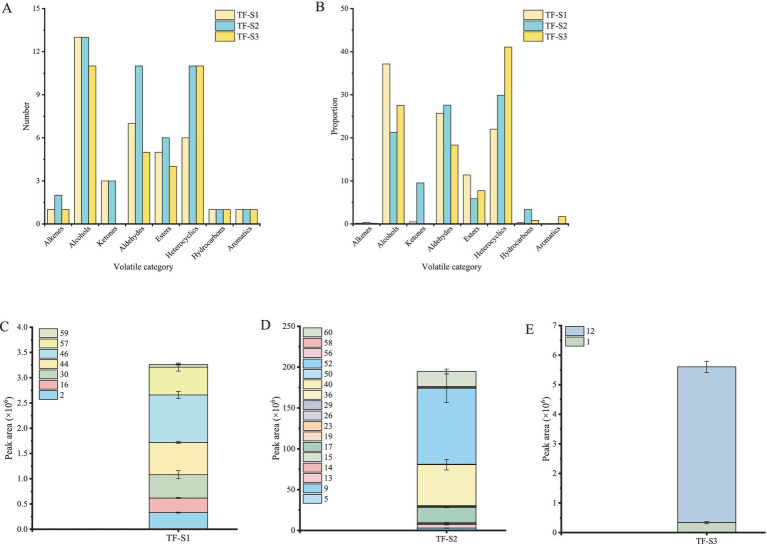
The volatile profiling of tea flowers during flowering. The number **(A)** and proportion **(B)** of volatile compounds in tea flowers. The volatile compounds uniquely identified in TF-S1 **(C)**, TF-S2 **(D)** and TF-S3 **(E)**. Compound nos. Correspond to Table 1.

Totally 63 volatile compounds were identified in tea flowers with different flowering stages of TF-S1 (38), TF-S2 (49), and TF-S3 (35) (Table 1). The identified components belonged to various chemical groups, including alcohols, aldehydes, alkenes, ketones, esters, heterocyclics, hydrocarbons and aromatics, as shown in Figure 1. The proportions and numbers of these volatile categories were different among the three samples (Figures 2A,B). Notably, alcohols, aldehydes and heterocyclics were the most abundant compounds, accounting for more than 18% of the total volatiles amount in each sample. With the advancement of flower growth, the proportions of alcohols and esters were diminished, whereas the heterocyclic compounds were enhanced. The peak proportion of heterocyclic compounds was observed in TF-S3, reaching to 41.06%, which was 1.87 and 1.38 times of that in TF-S1 and TF-S2, respectively. In addition, twenty-two volatile compounds were commonly found in all three samples, accounting for 34.92% of the total identified compounds. However, 7, 17, and 2 volatiles were uniquely identified in TF-S1, TF-S2, and TF-S3 samples, respectively (Figures 2C–E).

**Table 1:** The identified volatile compounds and their odor description of tea flowers during blooming.

No.	Volatile compounds	RT (min)	RI	RI-f	ID^ζ^	Threshold (mg/m^3^)^#^	Odor quality*^ψ^*	Relative amount (peak area, ×10^6^)		
								TF-S1	TF-S2	TF-S3
1	2-(Vinyloxy)ethanol	3.12	724	728	MS,RI	n.f.	——	nd	nd	0.34 ± 0.04
2	Acetoin*	3.21	729	718	MS,RI,S	14	Milky	0.33 ± 0.01	nd	nd
3	(*Z*)-2-Penten-1-ol	3.84	763	765	MS,RI	720	Green, fruity	7.22 ± 0.25	14.85 ± 2.13	7.44 ± 0.16
4	Hexanal*	4.55	801	801	MS,RI,S	0.23	Grassy, green, fresh, fatty	18.74 ± 0.98	44.47 ± 1.14	18.57 ± 1.86
5	2-Methyl-2-pentenal	5.05	815	810	MS,RI	290	Green, pungent, fruity	nd	0.34 ± 0.03	nd
6	Furfural*	5.57	829	833	MS,RI,S	2.8	Almond, caramel, honey, roasted, fatty	11.14 ± 0.83	52.82 ± 7.07	14.26 ± 0.51
7	(*E*)-2-Hexenal*	5.98	840	847	MS,RI,S	0.0031	Green, fresh, fruity	0.55 ± 0.02	0.57 ± 0.02	nd
8	2-Hexenal*	6.33	850	851	MS,RI,S	0.48	Grassy, herbal	34.18 ± 4.3	58.13 ± 0.32	27.43 ± 4.50
9	(*Z*)-3-Hexen-1-ol*	6.39	852	854	MS,RI,S	0.013	Green, grass, fruity	nd	2.57 ± 0.36	nd
10	(*Z*)-4-Hexen-1-ol*	6.42	853	857	MS,RI,S	100	Green, herbal, musty	0.40 ± 0.05	0.85 ± 0.05	0.60 ± 0.06
11	2-Furanmethanol*	6.65	859	859	MS,RI,S	32	Burnt, sweet, bready, caramel-like	nd	3.53 ± 0.27	2.64 ± 0.19
12	1,3-Dimethylbenzene	6.90	866	866	MS,RI	0.18	Plastic, aromatic	nd	nd	5.26 ± 0.19
13	1-Hexanol*	6.95	867	868	MS,RI,S	0.034	Green, grassy	nd	4.38 ± 0.70	nd
14	4-Cyclopentene-1,3-dione	7.37	879	880	MS,RI	n.f.	——	nd	1.37 ± 0.16	nd
15	3(2H)-Pyridazinone	7.43	880	885	MS,RI	n.f.	Burnt, smoky	nd	1.29 ± 0.14	nd
16	2-Heptanone	7.75	889	891	MS,RI	0.0035	Pear-like, fruity	0.29 ± 0.01	nd	nd
17	Heptanal*	8.26	903	907	MS,RI,S	0.26	Green, oily, grassy	nd	18.23 ± 0.30	nd
18	2-Heptanol	8.27	903	900	MS,RI	0.1	Leafy green, vegetable-like	18.47 ± 0.04	nd	21.24 ± 0.45
19	Methional	8.49	907	907	MS,RI	0.06	Creamy, earthy, vegetable, potato-like	nd	0.25 ± 0.02	nd
20	2-Acetylfuran	8.57	909	911	MS,RI	15025.2	Almond-like, nutty, cocoa-like	nd	1.84 ± 0.26	0.34 ± 0.04
21	2,5-Dimethylpyrazine*	8.85	915	917	MS,RI,S	1.82	Peanut, coffee, cocoa-like, nutty	nd	0.17 ± 0.01	1.88 ± 0.13
22	5-Methyl-2-furanmethanol	10.68	953	958	MS,RI	32	Roasted, meaty, baked potato-like	nd	3.64 ± 0.12	0.98 ± 0.04
23	5-Methyl-2-furancarboxaldehyde	11.00	960	961	MS,RI	500	Sweet, caramellic, bready, brown, coffee-like	nd	0.31 ± 0.03	nd
24	Benzaldehyde*	11.01	960	962	MS,RI,S	0.085	Almond-like, fruity, cherry-like, powdery, nutty	1.82 ± 0.13	3.85 ± 0.34	2.25 ± 0.30
25	2,4-Dihydroxy-2,5-dimethyl-3(2H)-furan-3-one	12.04	981	977	MS,RI	n.f.	——	3.19 ± 0.06	nd	5.06 ± 0.78
26	6-Methyl-5-hepten-2-one*	12.21	985	986	MS,RI,S	0.01889	Fruity, apple-like, citrus	nd	0.18 ± 0.03	nd
27	Hexanoic acid	12.23	985	982	MS,RI	0.0048	Acrid flavor	5.59 ± 0.09	8.22 ± 1.48	8.25 ± 1.27
28	2-Pentylfuran	12.40	989	989	MS,RI	0.019	Fruity, green, earthy, beany	1.75 ± 0.13	2.95 ± 0.23	2.07 ± 0.20
29	2-Methyl-6-hepten-1-ol	12.66	994	994	MS,RI	2000	Green, sweet	nd	1.06 ± 0.07	nd
30	6-Methyl-5-hepten-2-ol	12.68	995	993	MS,RI	2000	Coriander-like, green, sweet	0.46 ± 0.08	nd	nd
31	Octanal	13.13	1,004	1,004	MS,RI	0.17	Fatty, green, citrus, waxy	1.10 ± 0.10	3.13 ± 0.54	nd
32	(*E*)-3-Hexen-1-ol acetate	13.24	1,006	1,005	MS,RI	870	Fresh, green, fruity, sweet	13.17 ± 0.33	14.10 ± 1.95	9.55 ± 1.28
33	*N*-Acetyl-4(H)-pyridine	13.86	1,018	1,032	MS,RI	n.f.	——	1.03 ± 0.10	nd	1.39 ± 0.24
34	Limonene*	14.31	1,027	1,026	MS,RI,S	0.21	Citrus, lemon, orange-like, green	0.42 ± 0.06	1.26 ± 0.01	0.46 ± 0.06
35	Benzyl alcohol*	14.74	1,036	1,036	MS,RI,S	2546.21	Fruity, rose-like	2.79 ± 0.30	2.46 ± 0.03	0.83 ± 0.12
36	2-Methylphenol	14.90	1,039	1,044	MS,RI	0.0012	Musty, phenolic, plastic, medicinal, herbal	nd	0.42 ± 0.02	nd
37	Benzeneacetaldehyde*	15.09	1,043	1,045	MS,RI,S	6.3	Floral, rose, cherry-like	4.84 ± 0.28	1.26 ± 0.24	4.98 ± 0.04
38	1-Ethyl-1H-pyrrole-2-carboxaldehyde	15.28	1,046	1,046	MS,RI	65,000	Burnt, roasted, smoky	nd	2.13 ± 0.18	1.80 ± 0.16
39	*α*-Methylbenzenemethanol	16.01	1,061	1,063	MS,RI	5,100	Floral, light gardenia-like	2.29 ± 0.10	12.50 ± 2.04	3.11 ± 0.11
40	Acetophenone*	16.16	1,064	1,065	MS,RI,S	0.0012	Sweet, cherry pit, vanilla-like	nd	50.18 ± 6.46	nd
41	2-Acetylpyrrole*	16.20	1,064	1,064	MS,RI,S	>2	Nutty, musty	4.09 ± 0.08	0.45 ± 0.06	8.80 ± 1.69
42	Linalool oxide II*	16.49	1,070	1,074	MS,RI,S	60	Sweet, floral, creamy	2.16 ± 0.35	4.87 ± 0.08	1.94 ± 0.06
43	Linalool oxide I*	17.30	1,086	1,098	MS,RI,S	100	Sweet, floral, creamy	3.40 ± 0.07	8.44 ± 0.91	3.08 ± 0.05
44	2-Nonanone	17.56	1,091	1,092	MS,RI	0.032	Fruity, floral, fatty, herb	0.64 ± 0.02	nd	nd
45	Linalool*	18.02	1,100	1,104	MS,RI,S	0.0024	Floral, sweet, green	21.99 ± 1.32	47.67 ± 0.84	19.15 ± 2.68
46	Hotrienol*	18.17	1,103	1,101	MS,RI,S	110	Fresh, floral, fruity	0.94 ± 0.07	nd	nd
47	Nonanal*	18.27	1,105	1,104	MS,RI,S	0.0031	Floral, green, lemon-like	3.19 ± 0.44	18.75 ± 2.82	3.34 ± 0.19
48	Maltol	18.39	1,108	1,110	MS,RI	1,240	Caramel-like	nd	2.13 ± 0.19	1.73 ± 0.02
49	Phenylethyl alcohol*	18.56	1,111	1,116	MS,RI,S	0.012	Floral, rose-like	31.97 ± 3.25	12.77 ± 1.52	25.46 ± 0.30
50	Cosmene	19.42	1,129	1,130	MS,RI	n.f.	Herb, citrus	nd	0.55 ± 0.08	nd
51	Pyranone	20.07	1,142	1,151	MS,RI	n.f.	Hay-like	34.00 ± 3.16	nd	87.52 ± 10.20
52	2,3-Dihydro-3,5-dihydroxy-6-methyl-4H-pyran-4-one	20.15	1,143	1,154	MS,RI	n.f.	——	nd	92.99 ± 17.39	nd
53	3(*Z*)-Hexenyl butanoate	22.26	1,186	1,184	MS,RI	500	Fresh, green, fruity, vegetable-like	0.54 ± 0.02	0.87 ± 0	nd
54	Methyl salicylate*	22.42	1,189	1,192	MS,RI,S	0.016	Peppermint, wintergreen mint	9.42 ± 0.82	9.73 ± 1.39	3.49 ± 0.64
55	*α*-Terpineol*	22.65	1,194	1,191	MS,RI,S	0.86	Pleasant, floral	0.45 ± 0.01	0.89 ± 0.02	nd
56	Decanal*	23.26	1,207	1,205	MS,RI,S	0.0026	Sweet, citrus, waxy, floral	nd	0.77 ± 0.05	nd
57	Geraniol*	25.36	1,251	1,255	MS,RI,S	0.6	Rose-like, sweet, honey-like	0.55 ± 0.08	nd	nd
58	Methyl 2-methoxybenzoate	29.13	1,334	1,337	MS,RI	790	Hyacinth-like, herb	nd	1.48 ± 0.11	nd
59	2-(1,3-Butadienyl)-1,3,5-trimethylbenzene	29.84	1,350	1,373	MS,RI	n.f.	——	0.05 ± 0.01	nd	nd
60	6,10,14-Trimethyl-2-pentadecanone	48.87	1841	1846	MS,RI	n.f.	——	nd	18.17 ± 3.03	nd
61	Butyl hexadecanoate	59.86	2,184	2,188	MS,RI	>2000	Waxy	4.48 ± 0.50	4.52 ± 0.5	9.78 ± 1.50
62	Tricosane*	63.44	2,300	2,300	MS,RI,S	10,000,000	Alkane	0.78 ± 0.08	nd	2.59 ± 0.29
63	Butyl octadecanoate	65.63	2,371	2,382	MS,RI	>500	Waxy	0.89 ± 0.01	1.11 ± 0.02	1.03 ± 0.03
Total								250.74	543.04	308.64

RI, the retention index (RI) was computed by using n-alkanes (C5-C28) under the same chromatographic conditions with the detected volatile compounds; RI-f, the data was from the literature (http://webbook.nist.gov/chemistry/). Each value in the table was presented as the mean ± standard deviation (*n* = 3). The compounds indicated with “*” were identified using authentic standard compounds. TF-S1, unopened tea flowers; TF-S2, half-opened tea flowers; TF-S3, fully opened tea flowers. ^ζ^Identification method. MS, identification based on the NIST 2017 mass spectral database; RI, retention index; S, the compounds were identified using authentic standard compounds. ^#^All the odor thresholds were obtained from: a, “Odor & Flavor Detection Thresholds in Air (In Parts per Billion, lg/L)” (http://www.leffingwell.com/odourthre.htm). “n.f.”, data was not found in the literature. “——,” no odor description information was found in the literature. nd, not detectable.

#### 3.1.1 Volatile profiling of TF-S1

A total of 41 volatiles were detected in TF-S1, with 38 volatile compounds successfully identified (Table 1). The TF-S1 samples tended to contain the highest proportion of alcohols (37.13%), among which phenylethyl alcohol, linalool and 2-heptanol were the three most abundant compounds, accounting for 77.81% of the total alcohols detected. Phenylethyl alcohol imparting floral, rose-like odors (35), is the product of glycoside hydrolysis in tea (36, 37). 2-Heptanol having herbal flavor is derived from lipid degradation, with the unsaturated fatty acids such as palmitoleic acid and oleic acid as precursors (36). Linalool, a contributor for floral odor in teas, is recognized as the key aroma compound in all tea samples (38, 39). In addition, benzyl alcohol, linalool oxide I and linalool oxide II, which contribute to floral odors (36), were identified in TF-S1 but were of low abundance. Among the seven aldehydes identified in TF-S1, 2-hexenal and hexanal exhibiting green or grassy odors (38, 39), were the major volatile compounds. Particularly, the amount of 2-hexenal was significantly higher than the other aldehydes, nearly twice as much as hexanal. In addition to the two components and (*E*)-2-hexenal, the other four aldehydes, namely benzaldehyde, octanal, benzeneacetaldehyde and nonanal have floral or fruity odors (36), which are the important contributors to tea aroma. The two most abundant heterocyclic compounds were pyranone and furfural, comprising 81.78% of the total amounts of heterocyclic compounds. The former was detected in tea related products for the first time, while the latter was commonly identified in baked teas as a contributor of almond-like and caramel-like odors (30, 40, 41). Most of the identified esters had green or fatty flavor, in which (*E*)-3-hexen-1-ol acetate and methyl salicylate were the major compounds with high amounts. Methyl salicylate is considered as an important aroma compound for tea quality, and is mainly liberated by hydrolyzing glycosides in tea (38, 39, 42). Three ketones, including acetoin, were identified in TF-S1. Acetoin with pleasant milky odor (43) is rarely detected in previous tea samples. In addition, only one chemical compound with a relatively low amount, was identified in alkenes, hydrocarbons, and aromatics.

#### 3.1.2 Volatile profiling of TF-S3

Totally 35 volatiles were identified in TF-S3 using GC–MS technology (Table 1). In contrast to TF-S1, these identified components belonged to seven chemical groups, with no ketones detected in the sample. The volatile compositions, amounts, and proportions of volatile categories were different from those in TF-S1. Similarly, alcohols, aldehydes and heterocyclic compounds were the most abundant, accounting for 86.91% of the total identified volatiles, while alkenes were the least abundant (0.15%). Limonene was the sole alkene compound identified, and its amount did not significantly differ from that in TF-S1. In comparison to TF-S1, five heterocyclic compounds including 2-furanmethanol, 2-acetylfuran, 2,5-dimethylpyrazine, 5-methyl-2-furanmethanol and 1-ethyl-1H-pyrrole-2-carboxaldehyde were newly identified. The former two compounds have been found in large-leaf yellow tea under high intensity of roasting treatment (30), and the latter one exhibiting roasted or smoky notes (41) is generated in the final product *Shuixian* oolong tea after full fire processing (32). The formation of 5-methyl-2-furanmethanol is in relation to Maillard reaction from D-glucose and L-theanine (32), and 2,5-dimethylpyrazine, which imparts characteristic roasted peanutty flavor, is considered as the key aroma compound in tea (32). In terms of the common volatiles in TF-S1 and TF-S3, heterocyclic compounds were still dominated by pyranone, accounting for 69.05% of the total amounts of this group in TF-S3. Additionally, the amounts of these common volatile compounds, except for 2-acetylpyrrole, raised significantly as the tea flower progressed from stage I to stage III. A total of 11 alcohols were identified in the sample, of which 2-(vinyloxy)ethanol and maltol absent in TF-S1. Maltol having caramel-like odor, which is an important flavor ingredient detected in Japanese soy sauce (44), was not found in previous tea samples. Meanwhile, trace amounts of alcohols in TF-S1, such as 6-methyl-5-hepten-2-ol, hotrienol and geraniol, which are identified in Wuyi rock tea (31), were not detected in TF-S3. Although phenylethyl alcohol, linalool and 2-heptanol remained the three most abundant alcohols in TF-S3, the amounts of the former two volatiles dropped compared to TF-S1. With the flowers development (from stage I to stage III), the amounts of aldehydes and esters with green flavor were decreased, whereas the aldehydes with floral odors or the esters with fatty notes were enhanced. Octanal, (*E*)-2-hexenal and 3(*Z*)-hexenyl butanoate were not found in TF-S3.

#### 3.1.3 Volatile profiling of TF-S2

In total, 52 volatile compounds were detected in TF-S2, with 49 volatiles being identified (Table 1). Among these, alcohols (21.26%), aldehydes (27.58%) and heterocyclic compounds (29.85%) comprised 78.69% of the total amount. The number and amounts of identified volatiles in TF-S2 were much higher than those of TF-S1 and TF-S3. Moreover, the proportions or amounts of alkenes, ketones and hydrocarbons were enhanced significantly. The amount of limonene, a representative component common to all three samples, was almost three times higher in TF-S2 compared to the other two samples. Cosmene, an unsaturated alkene commonly found in herbal essential oils (45) and a bound-form volatile compound of *Rubus corchorifolius* fruit (46), was detected in tea flowers firstly. Different from TF-S1, three new ketones were identified in TF-S2, with 6-methyl-5-hepten-2-one being the least amount. This compound is identified in oolong tea as a carotenoid-derived aroma compound (36, 47). Acetophenone imparts sweet, or citrus flavor, which is derived from L-phenylalanine in tea flowers (48), was also found in tea leaves (49). It was identified in high abundance only in TF-S2, differing slightly from the results of Joshi et al. (50) due to variations in location, variety, sample status, and aroma extraction methods of the tea flowers. Although the largest number of esters were identified in TF-S2, their proportions significantly decreased from stage I to stage II. Among them, (*E*)-3-hexen-1-ol acetate and methyl salicylate remained the two compounds with the highest amounts. Similar to esters, the proportions of alcohols first decreased and then increased with the advancement of flower growth, with the lowest observed in TF-S2. However, the amounts of alcohols imparting floral flavor, such as linalool, linalool oxide I and linalool oxide II, increased significantly. These are aroma-active compounds in tea, with linalool being a vital contributor of the floral odor in tea (36), and the most abundant alcohols in TF-S2. In practice, linalool has a floral odor with green flavor, which might be the main source of floral and green notes in the sensory evaluation of TF-S2. Four aldehydes, namely 2-methyl-2-pentenal, heptanal, methional and decanal were only identified in TF-S2. Heptanal and decanal are produced by lipid oxidation in tea (36), and contribute to the fruity or floral flavor of tea flowers (36, 51). Methional, with a low threshold value (0.06 ppb in air), is usually recognized as the precursor of methanethiol (52). Among the common aldehydes, the amounts of hexanal, 2-hexenal, benzaldehyde, and nonanal were significantly higher in TF-S2 than those of the other two samples, while benzeneacetaldehyde presented an opposite pattern.

Eleven heterocyclics were identified in TF-S2, among which furfural, 2-phetylfuran and 2-acetylpyrrole were the common heterocyclics in three tea flower samples. Furfural and 2-phetylfuran were most abundant in TF-S2, whereas TF-S2 had the least amount of 2-acetylpyrrole. In comparison to TF-S3, TF-S2 had the significantly lower amounts of 2,5-dimethylpyrazine but much higher amounts of 2-acetylfruan, 5-methyl-2-furanmethanol and 2-furanmethanol. 5-Methyl-2-furancarboxaldehyde, which has been found in large-leaf yellow tea and oolong tea after high intensity of roasting treatment (30, 32) together with 3(2H)-pyridazinone were solely identified in TF-S2. In addition, hexanoic acid with acrid odor (53) was identified in all samples. The amounts of hexanoic acid gradually increased with the development of blooming, while their proportion first declined and then raised during flowering, being lowest in TF-S2 (1.51%) and highest in TF-S3 (2.67%).

As the flowering stage progressed, TS-S2 possessed a significantly greater number and amounts of volatiles, most of which had floral and fruity odors as well as the pleasant flavor. Their amounts had a significant increasement compared with the other two tea flower samples. Meanwhile, some volatile compounds with green or fatty odors decreased or volatilized during flowering. These findings were in line with those of Joshi et al. (50), who observed that half-opened tea flowers had the most volatiles, and the highest levels of linalool, linalool oxide I & II, benzaldehyde, and acetophenone, which was only identified in TF-S2.

### 3.2 Principal component analysis

**Figure 3:**
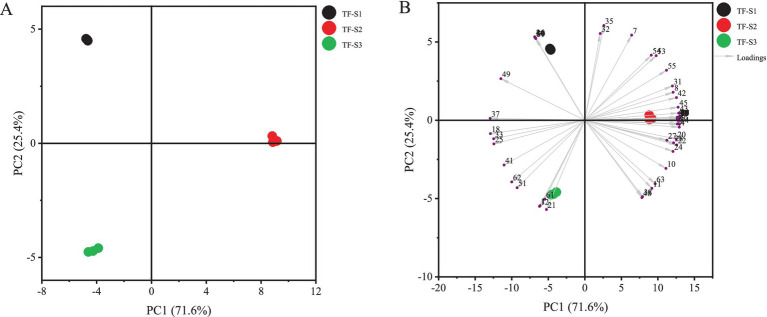
PCA analysis of tea flowers in different flowering stages. The score plot **(A)** and biplot **(B)** of PCA analysis. Compound nos. Correspond to Table 1.

To further understand intuitively the aroma characteristics of tea flowers and distinguish the differences among tea flowers from various flowering stages, the PCA was conducted on the identified volatiles (Figure 3). As can be seen in Figure 3A, the accumulated contribution rate of the first two principal components (PC 1 for 71.6%, PC 2 for 25.4%) was 97.0%, which appeared to represent the sufficient information of tea flower samples. Notably, the three samples were well separated according to the flowering stages. Specifically, TF-S1 exhibited high scores on the negative PC 1 and positive PC 2, where the loadings of characteristic volatile compounds including hotrienol, geraniol, 2-nonanone, 6-methyl-5-hepten-2-ol, acetoin, 2-(1,3-butadienyl)-1,3,5-trimethylbenzene were prominent. These compounds, along with phenylethyl alcohol and benzeneacetaldehyde, were uniquely identified in the initial stages of florescence of tea flower. This suggests that the different aroma properties of TF-S1 compared to TF-S2 or TF-S3 might be attributed to its uniquely volatile compounds. On the other hand, TF-S3 showed high scores on both the negative PC 1 and negative PC 2, containing high loadings of compounds, such as 2,5-dimethylpyrazine, 1,3-dimethylbenzene, butyl hexadecanoate, 2-acetylpyroole, tricosane, pyranone. Among these, pyranone was the most abundant aroma compound in TF-S3 of the late stages of tea flowering (Figure 3B). In addition, the levels of benzeneacetaldehyde, 2-heptanol, *N*-acetyl-4(H)-pyridine, and 2,4-dihydroxy-2,5-dimethyl-3(2H)-furan-3-one were similar in TF-S1 and TF-S3, suggesting they were co-characteristic aroma compounds in both samples. TF-S2 scored high on the positive PC 1, which contained high loadings of benzyl alcohol, (*E*)-3-hexen-1-ol acetate, 3(*Z*)-hexenyl butanoate, linalool oxide II, linalool, linalool oxide I, benzaldehyde, furfural, 2-acetylfuran, 5-methyl-2-furanmethanol, 2-pentylfuran, 2-furanmethanol, maltol. These characteristic volatiles with floral, fruity, almond-like odors, along with heterocyclic compounds, were of most abundance in the half-opened tea flowers. The PCA results indicated that the fully and half-opened tea flowers possessed large amounts of volatiles with floral or fruity odors and heterocyclic compounds, whereas volatile compounds imparting green or fatty odors were abundant in the initial stages of tea flower blossoming.

However, it is important to note that the aroma impact of a component depends on its concentration and odor threshold value. Therefore, a comprehensive analysis of the aroma contributions of volatile compounds in tea flowers is necessary, and the actual aroma profile of tea flowers should be assessed from a sensorial point of view.

### 3.3 Odor profiles of volatiles in tea flowers during blooming

The contribution of an individual component to the overall aroma of tea flowers is determined by its aroma character impact (ACI). The ACI values of aroma compounds in tea flowers were calculated based on the threshold data from reported references, and the ACI of identified volatiles is presented in Supplementary Table S2. Totally, 8 volatile compounds had ACI values higher than one in three samples, of which 7, 5, and 6 volatiles with ACI greater than one were identified in TF-S1, TF-S2, and TF-S3, respectively (Table 2). Generally, these aroma character impact molecules mainly imparted floral, sweet or green notes, contributing 97.49, 97.78, and 97.68% to the aroma of TF-S1, TF-S2, and TF-S3, respectively (Table 2).

**Table 2:** The identified volatiles with ACI above one in tea flowers during blooming.

Volatile compounds	ACI (%)		
	TF-S1	TF-S2	TF-S3
(*E*)-2-Hexenal	1.16	0.25	nd
2-Heptanol	1.20	nd	1.56
Hexanoic acid	7.58	2.35	12.60
Acetophenone	nd	57.35	nd
Linalool	59.66	27.24	58.48
Nonanal	6.70	8.30	7.90
Phenylethyl alcohol	17.35	1.46	15.55
Methyl salicylate	3.83	0.83	1.60

TF-S1, unopened tea flowers; TF-S2, half-opened tea flowers; TF-S3, fully opened tea flowers. nd, not detectable.

Among the identified volatiles, linalool was the only volatile compound with an ACI above 27 in all samples. It also made the most significant contribution to the aroma character in TF-S1 and TF-S3, indicating that it had the positive influence on floral or green notes in tea flower samples, consistent with the findings from Gao et al. (54) that linalool has been reported to be the potential key aroma compound in tea flowers from albino cultivars with the highest relative abundance. In contrast, for TF-S2, acetophenone imparting sweet odor was the most prominent aroma contributor (ACI = 57.35). Nonanal, which contributes floral or green odors, had the third highest ACI value of 8.30, whereas the remaining compounds played a much less important role in the aroma contribution. In comparison to TF-S2, acetophenone was not found in TF-S1, where phenylethyl alcohol (ACI = 17.35), hexanoic acid (ACI = 7.58), (*E*)-2-hexenal (ACI = 1.16), and methyl salicylate (ACI = 3.83) had significantly higher ACI values. Similarly to TF-S1, the volatiles phenylethyl alcohol (ACI = 15.50), hexanoic acid (ACI = 12.60), nonanal (ACI = 7.90), and methyl salicylate (ACI = 1.60) were notable contributors for TF-S3. However, (*E*)-2-hexenal with green flavor was not observed in TF-S3. Notably, Cui et al. (55) discovered that acetophenone is the most abundant volatile compound in tea flower at the fully-opened stage. The significant different might be due to the distinct tea cultivar utilized and the different aroma extraction techniques employed.

### 3.4 Sensory profiles of tea flowers during flowering

**Figure 4:**
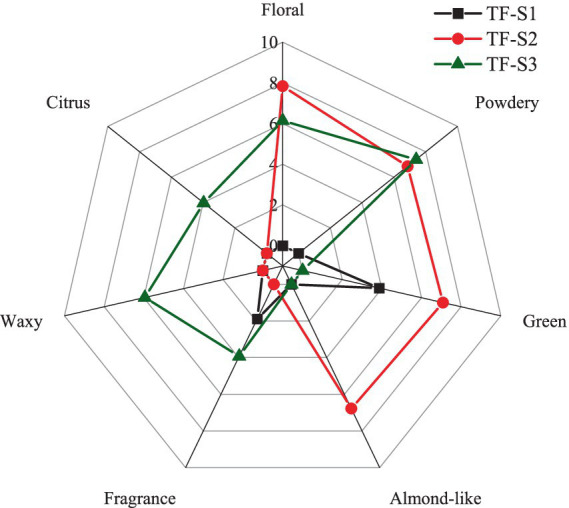
The radar profile of sensory aroma attributes of tea flowers during blooming.

The radar profile illustrating the sensory aroma attributes of tea flowers during flowering stage is shown in Figure 4. The detailed scores on intensity of aroma attributes were shown in Supplementary Table S3. Only moderate green and weak fragrance odors were recorded in TF-S1, while TF-S3 had extremely high intensity of powdery odor, coupled with strong floral and waxy odors, as well as moderate citrus and fragrance notes. Compared to TF-S3, TF-S2 possessed higher intensity of floral odor and lower intensity of powdery odor. Notably, TF-S2 also featured a special characteristic of almond-like odor with high intensity, potentially attributed to the abundant presence of furfural, which imparts such an aroma. As for the green flavor in TF-S2, it differed from that of noted in TF-S1, accompanied with the floral odor, possibly originating from linalool. However, further research is required to ascertain the exact contribution of linalool to the floral and green notes of half-opened tea flowers. The green flavor was not noted in TF-F3 might be associated with the absence of (*E*)-2-hexenal, which has been considered a positive contributor to green odor in tea flower (54). Practical sensory evaluation results revealed that half-opened and fully-opened tea flowers had different aroma characteristics, the latter exhibited powdery odor, whereas, the former was dominated by floral note, both with extremely high intensities. Linalool and phenylethyl alcohol might be the potential contributors of powdery odor in TF-S3, while acetophenone and linalool contributed to the floral note in TF-S2. The unique aroma characteristics of tea flowers at different stages of flowering could be harnessed to create distinctive and appealing products. The TF-S2 had strong floral and almond-like odors, with sensory scores of 7.84 and 6.77, respectively, making it an ideal choice for use in perfumes and cosmetics to impart a fresh and inviting scent. Similarly, the powdery odor of TF-S3, with a sensory score of 7.39, could add a unique and intriguing note to beverages and other products. Further research is encouraged to explore the full potential of tea flowers in these and other applications.

## 4 Conclusion

In the present study, the volatile compositions and aroma profiles of tea flowers at different developmental stages were analyzed using HS-GC–MS technology, complemented by ACI determination and sensory evaluation. The volatiles in tea flowers were dominated by alcohols, aldehydes and heterocyclic compounds, with variations across stages. TF-S2 tended to contain higher proportions of aldehydes, ketones, hydrocarbons, and alkenes, but lower alcohols and esters levels compared to flower buds and fully opened tea flowers. Eight volatiles, including linalool (ACI >27 in all samples) and acetophenone (unique to TF-S2), were the key aroma compounds. Half-opened tea flowers exhibited strong floral odor and the characteristic almond-like flavor, while fully opened tea flowers had strong powdery and moderate floral odors. Overall, half-opened or fully-opened tea flowers had higher amounts of total volatiles and superior aroma characteristics. These findings provide the theoretical basis for the understanding the aroma profiles and application performance of tea flowers, suggesting further research into their physicochemical composition, bioactivity, and the practical application in functional foods, beverages, and food flavor industries.

## In memoriam

We would like to dedicate this article with profound affection to the memory of first author’s mother, who passed away at the end of September 2024. She provided many valuable opinions and suggestions for the shaping of this article, and it is due to her constant encouragement and support that the author has been able to persist in the scientific research work.

## Data Availability

The original contributions presented in the study are included in the article/Supplementary material, further inquiries can be directed to the corresponding authors.

## Footnotes

^1^ https://www.chiplot.online/
