# Supplementary material for: Characterization of the volatile compounds in tea (Camellia sinensis L.) flowers during blooming
Source: Front Nutr. 2025 Jan 14;11:1531185. doi: 10.3389/fnut.2024.1531185 (PMC11772201; doi:10.3389/fnut.2024.1531185)
Supplement: Supplementary file 1 [file Table_1.docx]

**Supplementary material**

**Characterization of the volatile compounds in tea (*Camellia sinensis* L.) flowers during blooming**

(Abbreviated running title: Aroma profiles of tea flower)

Xiangyang Guo^a,b,c,*^, Lulu Wang^d^, Xiuting Huang^a^, Qiying Zhou^a,c,*^

*^a^ College of Tea and Food Science, Xinyang Normal University, Xinyang 464000, China*

*^b^ College of Chemistry and Environmental Engineering, Shenzhen University, Shenzhen 518060, China*

*^c^ Dabie Mountain Laboratory, Xinyang 464000, China*

*^d^ College of Horticulture, Shanxi Agricultural University, Taigu 030801, China*

*** Corresponding author:**

Xiangyang Guo, College of Chemistry and Environmental Engineering, Shenzhen University, Shenzhen 518060, China; E-mail address: xiangyang.guo@ahau.edu.cn.

Qiying Zhou, College of Tea and Food Science, Xinyang Normal University, 237 Nanhu Road, Xinyang 464000, China; E-mail address: zhouqy@xynu.edu.cn.

| **Table S1** The related chemical compounds used in this research | | | | |
| --- | --- | --- | --- | --- |
| Chemicals | Related information | | | |
|  | Purity | Supplier | City | Country |
| Acetoin | ≥95% | Sigma Aldrich | Shanghai | China |
| Linalool oxides (Ⅰ, Ⅱ, Ⅲ, Ⅳ) | ≥97% | Sigma Aldrich | Shanghai | China |
| Hexanal | >98% | Sigma Aldrich | Shanghai | China |
| (*E*)-2-Hexenal | ≥97% | Sigma Aldrich | Shanghai | China |
| 2-Hexenal | ≥95% | Sigma Aldrich | Shanghai | China |
| (*Z*)-3-Hexen-1-ol | >98% | Sigma Aldrich | Shanghai | China |
| 1-Hexanol | ≥99% | Sigma Aldrich | Shanghai | China |
| Heptanal | ≥95% | Sigma Aldrich | Shanghai | China |
| Benzaldehyde | ≥99.0% | Sigma Aldrich | Shanghai | China |
| Benzyl alcohol | ≥98% | Sigma Aldrich | Shanghai | China |
| Linalool | >95% | Sigma Aldrich | Shanghai | China |
| Nonanal | >95% | Sigma Aldrich | Shanghai | China |
| Phenylethyl alcohol | ≥99% | Sigma Aldrich | Shanghai | China |
| Methyl salicylate | ≥98% | Sigma Aldrich | Shanghai | China |
| *α*-Terpineol | ≥96% | Sigma Aldrich | Shanghai | China |
| Geraniol | ≥98% | Sigma Aldrich | Shanghai | China |
| Acetophenone | ≥98% | Sigma Aldrich | Shanghai | China |
| 2-Acetylpyrrole | >99% | Sigma Aldrich | Shanghai | China |
| 6-Methyl-5-hepten-2-one | ≥98% | Sigma Aldrich | Shanghai | China |
| Decanal | ≥98% | Sigma Aldrich | Shanghai | China |
| (*Z*)-4-Hexen-1-ol | >95% | Aladdin Chemicals | Shanghai | China |
| Furfural | >99.5% | Aladdin Chemicals | Shanghai | China |
| 2-Furanmethanol | >98% | Aladdin Chemicals | Shanghai | China |
| 2,5-Dimethylpyrazine | >98% | Aladdin Chemicals | Shanghai | China |
| Limonene | >95% | Aladdin Chemicals | Shanghai | China |
| Benzeneacetaldehyde | >95% | Aladdin Chemicals | Shanghai | China |
| Hotrienol | ≥98.0% | ChemFaces | Wuhan | China |
| *n*-alkanes (C5-C28) | Analytical grade | Supelco | Bellefonte, PA | USA |

| **Table S2** The aroma character impact (ACI) values of identified volatiles in tea flowers during blooming | | | | |
| --- | --- | --- | --- | --- |
| No. | Volatile compounds | ACI (%) | | |
|  |  | TF-S1 | TF-S2 | TF-S3 |
| 1 | 2-(Vinyloxy)ethanol | nd | nd | nd |
| 2 | Acetoin | 0.0002 | nd | nd |
| 3 | (*Z*)-2-Penten-1-ol | 0.0001 | 0.0000 | 0.0001 |
| 4 | Hexanal | 0.5306 | 0.2652 | 0.5918 |
| 5 | 2-Methyl-2-pentenal | nd | 0.0000 | nd |
| 6 | Furfural | 0.0259 | 0.0259 | 0.0373 |
| 7 | (*E*)-2-Hexenal | 1.1553 | 0.2522 | nd |
| 8 | 2-Hexenal | 0.4637 | 0.1661 | 0.4188 |
| 9 | (*Z*)-3-Hexen-1-ol | nd | 0.2711 | nd |
| 10 | (*Z*)-4-Hexen-1-ol | 0.0000 | 0.0000 | 0.0000 |
| 11 | 2-Furanmethanol | nd | 0.0002 | 0.0006 |
| 12 | 1,3-Dimethylbenzene | nd | nd | 0.2142 |
| 13 | 1-Hexanol | nd | 0.1767 | nd |
| 14 | 4-Cyclopentene-1,3-dione | nd | nd | nd |
| 15 | 3(2H)-Pyridazinone | nd | nd | nd |
| 16 | 2-Heptanone | 0.5395 | nd | nd |
| 17 | Heptanal | nd | 0.0962 | nd |
| 18 | 2-Heptanol | 1.2027 | nd | 1.5568 |
| 19 | Methional | nd | 0.0057 | nd |
| 20 | 2-Acetylfuran | nd | 0.0000 | 0.0000 |
| 21 | 2,5-Dimethylpyrazine | nd | 0.0001 | 0.0076 |
| 22 | 5-Methyl-2-furanmethanol | nd | 0.0002 | 0.0002 |
| 23 | 5-Methyl-2-furancarboxaldehyde | nd | 0.0000 | nd |
| 24 | Benzaldehyde | 0.1394 | 0.0621 | 0.1940 |
| 25 | 2,4-Dihydroxy-2,5-dimethyl-3(2H)-furan-3-one | nd | nd | nd |
| 26 | 6-Methyl-5-hepten-2-one | nd | 0.0131 | nd |
| 27 | Hexanoic acid | 7.5834 | 2.3487 | 12.5973 |
| 28 | 2-Pentylfuran | 0.5998 | 0.2129 | 0.7985 |
| 29 | 2-Methyl-6-hepten-1-ol | nd | 0.0000 | nd |
| 30 | 6-Methyl-5-hepten-2-ol | 0.0000 | nd | nd |
| 31 | Octanal | 0.0421 | 0.0253 | nd |
| 32 | (*E*)-3-Hexen-1-ol acetate | 0.0001 | 0.0000 | 0.0001 |
| 33 | *N*-Acetyl-4(H)-pyridine | nd | nd | nd |
| 34 | Limonene | 0.0130 | 0.0082 | 0.0161 |
| 35 | Benzyl alcohol | 0.0000 | 0.0000 | 0.0000 |
| 36 | 2-Methylphenol | nd | 0.4800 | nd |
| 37 | Benzeneacetaldehyde | 0.0050 | 0.0003 | 0.0058 |
| 38 | 1-Ethyl-1H-pyrrole-2-carboxaldehyde | nd | 0.0000 | 0.0000 |
| 39 | *α*-Methylbenzenemethanol | 0.0000 | 0.0000 | 0.0000 |
| 40 | Acetophenone | nd | 57.3515 | nd |
| 41 | 2-Acetylpyrrole | 0.0133 | 0.0003 | 0.0322 |
| 42 | Linalool oxide Ⅱ | 0.0002 | 0.0001 | 0.0002 |
| 43 | Linalool oxide Ⅰ | 0.0002 | 0.0001 | 0.0002 |
| 44 | 2-Nonanone | 0.1302 | nd | nd |
| 45 | Linalool | 59.6631 | 27.2414 | 58.4821 |
| 46 | Hotrienol | 0.0001 | nd | nd |
| 47 | Nonanal | 6.7007 | 8.2953 | 7.8968 |
| 48 | Maltol | nd | 0.0000 | 0.0000 |
| 49 | Phenylethyl alcohol | 17.3482 | 1.4595 | 15.5504 |
| 50 | Cosmene | nd | nd | nd |
| 51 | Pyranone | nd | nd | nd |
| 52 | 2,3-Dihydro-3,5-dihydroxy-6-methyl-4H-pyran-4-one | nd | nd | nd |
| 53 | 3(*Z*)-Hexenyl butanoate | 0.0000 | 0.0000 | nd |
| 54 | Methyl salicylate | 3.8337 | 0.8340 | 1.5987 |
| 55 | *α*-Terpineol | 0.0034 | 0.0014 | nd |
| 56 | Decanal | nd | 0.4062 | nd |
| 57 | Geraniol | 0.0060 | nd | nd |
| 58 | Methyl 2-methoxybenzoate | nd | 0.0000 | nd |
| 59 | 2-(1,3-Butadienyl)-1,3,5-trimethylbenzene | nd | nd | nd |
| 60 | 6,10,14-Trimethyl-2-pentadecanone | nd | nd | nd |
| 61 | Butyl hexadecanoate | 0.0000 | 0.0000 | 0.0000 |
| 62 | Tricosane | 0.0000 | nd | 0.0000 |
| 63 | Butyl octadecanoate | 0.0000 | 0.0000 | 0.0000 |
| TF-S1, unopened tea flowers; TF-S2, half-opened tea flowers; TF-S3, fully opened tea flowers.  nd, not detectable. | | | | |

| **Table S3** The detailed score on each aroma attribute of tea flower samples | | | | | |  |  |  |  |  |  |
| --- | --- | --- | --- | --- | --- | --- | --- | --- | --- | --- | --- |
| **FT-S1** | Score | | | | | | | | | | |
| Attribute | 1 | 2 | 3 | 4 | 5 | 6 | 7 | 8 | 9 | 10 | **Average** |
| Floral | 0 | 0 | 0 | 0 | 0 | 0 | 0 | 0 | 0 | 0 | 0 |
| Powdery | 0 | 0 | 0 | 0 | 0 | 0 | 0 | 0 | 0 | 0 | 0 |
| Green | 4 | 3.8 | 3.8 | 3.6 | 4.2 | 4.1 | 3.8 | 3.9 | 3.6 | 3.9 | 3.87 |
| Almond-like | 0 | 0 | 0 | 0 | 0 | 0 | 0 | 0 | 0 | 0 | 0 |
| Fragrance | 1.8 | 1.9 | 2 | 2.1 | 2 | 1.8 | 1.7 | 1.9 | 1.9 | 1.8 | 1.89 |
| Waxy | 0 | 0 | 0 | 0 | 0 | 0 | 0 | 0 | 0 | 0 | 0 |
| Citrus | 0 | 0 | 0 | 0 | 0 | 0 | 0 | 0 | 0 | 0 | 0 |
| **TF-S2** | Score | | | | | | | | | | |
| Attribute | 1 | 2 | 3 | 4 | 5 | 6 | 7 | 8 | 9 | 10 | **Average** |
| Floral | 8 | 7.9 | 8.1 | 7.8 | 7.6 | 7.9 | 7.8 | 8 | 7.6 | 7.7 | 7.84 |
| Powdery | 6.6 | 6.8 | 6.9 | 7 | 6.9 | 6.8 | 6.9 | 7 | 7 | 6.7 | 6.86 |
| Green | 7.2 | 7.3 | 7.2 | 7.4 | 6.8 | 6.9 | 7 | 7.1 | 6.9 | 7 | 7.08 |
| Almond-like | 6.9 | 6.8 | 6.8 | 6.8 | 6.7 | 6.6 | 6.8 | 6.8 | 6.8 | 6.7 | 6.77 |
| Fragrance | 0 | 0 | 0 | 0 | 0 | 0 | 0 | 0 | 0 | 0 | 0 |
| Waxy | 0 | 0 | 0 | 0 | 0 | 0 | 0 | 0 | 0 | 0 | 0 |
| Citrus | 0 | 0 | 0 | 0 | 0 | 0 | 0 | 0 | 0 | 0 | 0 |
| **TF-S3** | Score | | | | | | | | | | |
| Attribute | 1 | 2 | 3 | 4 | 5 | 6 | 7 | 8 | 9 | 10 | **Average** |
| Floral | 6 | 6.2 | 6.1 | 6 | 6.3 | 6.2 | 6.1 | 6 | 6.3 | 6.2 | 6.14 |
| Powdery | 7.6 | 7.5 | 7.9 | 7.1 | 7.2 | 7.3 | 7.4 | 7.2 | 7.1 | 7.6 | 7.39 |
| Green | 0 | 0 | 0 | 0 | 0 | 0 | 0 | 0 | 0 | 0 | 0 |
| Almond-like | 0 | 0 | 0 | 0 | 0 | 0 | 0 | 0 | 0 | 0 | 0 |
| Fragrance | 4 | 3.9 | 3.9 | 3.8 | 3.8 | 3.9 | 4 | 3.8 | 4.1 | 4 | 3.92 |
| Waxy | 6 | 6.1 | 6.2 | 5.7 | 5.9 | 5.8 | 6 | 6.1 | 6 | 5.7 | 5.95 |
| Citrus | 3.9 | 3.8 | 4 | 4.1 | 4.2 | 3.8 | 3.9 | 3.9 | 4 | 4.1 | 3.97 |
